# Draft Genome Sequence of Lichen-Forming Fungus *Caloplaca flavorubescens* Strain KoLRI002931

**DOI:** 10.1128/genomeA.00678-13

**Published:** 2013-08-29

**Authors:** Sook-Young Park, Jaeyoung Choi, Jung A. Kim, Nan-Hee Yu, Soonok Kim, Sergii Y. Kondratyuk, Yong-Hwan Lee, Jae-Seoun Hur

**Affiliations:** Korean Lichen Research Institute, Sunchon National University, Suncheon, South Koreaa; Department of Agricultural Biotechnology, Fungal Bioinformatics Laboratory, Center for Fungal Genetic Resources, and Center for Fungal Pathogenesis, Seoul National University, Seoul, South Koreab; Wildlife Genetic Resources Center, National Institute of Biological Resources, Incheon, South Koreac; M. H. Kholodny Institute of Botany, Kiev, Ukrained

## Abstract

Here we report a draft genome sequence of *Caloplaca flavorubescens* strain KoLRI002931, isolated from the bark of a gingko tree at Mt. Deogyu, Muju, South Korea. The genome sequence is 34,455,815 bp, with a GC content of 41.89%, consisting of 36 scaffolds.

## GENOME ANNOUNCEMENT

Although major fungal lineages may be derived from lichen symbiotic ancestors (1), little information is publicly available on the whole-genome sequences of lichen-forming fungi. Lichens have tremendous potential to produce very useful metabolites, with antitumor, antibacterial, antifungal, antiviral, anti-inflammatory, and antioxidant activities (2). These metabolites would be synthesized by various genes, including polyketide synthase (PKS) genes, but none has been functionally characterized (3).

The genus *Caloplaca* is cosmopolitan and is the largest genus of lichen-forming fungi identified to date. Their distribution ranges over all latitudes, including subpolar regions, and a wide range of altitudes. Eastern Asia is believed undoubtedly to be a hot spot of speciation for this genus, because more than 70 *Caloplaca* species have been reported from this region. Extensive surveys and investigations spanning many years led to the description of many new taxa (4–8). *Caloplaca flavorubescens* (Huds.) J. R. Laundon was selected as a model taxon for genomic investigation in lichen-forming fungi (9).

*C. flavorubenscens* was isolated from gingko tree bark at Mt. Deogyu (35°53′25.9″N, 127°46′44.6″E), Muju, South Korea, in 2005. DNA from axenic culture of the fungus was extracted using a DNeasy minikit (Qiagen, Valencia, CA). Sequencing was performed using a whole-genome shotgun strategy with an Illumina HiSeq2000 (Macrogen, Inc., Seoul, South Korea). The total size of the assembled genome of *C. flavorubescens* KoLRI002931 was 34,455,815 bp, with a GC content of 41.89%, representing 541-fold coverage. The short reads were assembled using ALLPATHS-LG (10), yielding 36 scaffolds (≥1,000 bp) generated from 189 contigs. Gene prediction was performed by using MAKER (11), producing 9,695 protein-coding sequences. According to three gene family pipelines (12–14), 235 transcription factor genes, 92 cytochrome P450 genes, and 1,812 genes encoding secretory proteins were predicted. In addition, 13 putative PKS genes, containing ketoacyl synthase, acyltransferase, and acyl carrier domains, were predicted by a domain search (15).

Further analysis of the genome, including functional and biochemical analyses, would provide more information on fungal symbiosis and secondary metabolism. In addition, the genome sequence of *C. flavorubescens* will facilitate comparative genomics with other lichen-forming fungi as well as species in the phylum *Ascomycota*.

### Nucleotide sequence accession numbers. 

The draft genome sequence of *C. flavorubescens* KoLRI002931 has been deposited in DDBJ/EMBL/GenBank under accession no. AUPK00000000. The version described in this article is the first version, accession no. AUPK01000000.
